# Electrification and Renewable Energy Generation

**DOI:** 10.1155/2015/275238

**Published:** 2015-03-26

**Authors:** Linni Jian, Hua Bai, Wenxiang Zhao, Jianing Liang

**Affiliations:** ^1^Department of Electrical and Electronic Engineering, South University of Science and Technology of China, Shenzhen 518055, China; ^2^Department of Electrical and Computer Engineering, Kettering University, 1700 University Avenue, Flint, MI 48504, USA; ^3^School of Electrical and Information Engineering, Jiangsu University, No. 301, Xuefu Road, Zhenjiang 212013, China; ^4^Shenzhen Institutes of Advanced Technology, Chinese Academy of Sciences, 1068 Xueyuan Avenue, Shenzhen University Town, Shenzhen 518055, China

With increasing worldwide concerns on energy crisis and global warming, the topics on both electrification and renewable energy generation have become very attractive most recently. In our opinion, electrification means higher energy efficiency and thereby effective energy saving, while renewable energy generation means independence of fossil energy and thereby zero emission. Consequently, electrification plus renewable energy generation points a promising way to environmental and sustainable development of human beings. Nevertheless, we are still facing critical challenges in many aspects, such as how to harvest and utilize renewable energy in a high-efficient and low-costly way and how to process and convert electrical energy so as to better fulfill the demands in practical applications.

The main objective of this special issue is to bring together researchers pursuing these fields and present their most recent working progress. After critical peer review, several papers are selected for publication in this issue, which cover many significant aspects related to the topic of electrification and renewable energy generation. L. Xu et al. proposed a novel linear fault-tolerant permanent-magnet machine, which can be applied to high-efficient urban rail transit systems. Y. Fan et al. investigated a new flux-modulated brushless drive motor for electric vehicles. Q. Zhang et al. proposed a novel pulse-wide modulation method to reduce current ripples of Z-source inverters. H. Li et al. presented an effective multisource energy harvesting system for wireless sensor nodes. In order to solve problems arising from wide-area backup protection, Z. Zhang et al. proposed a novel protection algorithm. J. Si et al. reported a tubular linear generator for harvesting wave energy. G. S. B. Ganandran et al. reported the result of an investigation on the potential energy saving of the lighting systems at selected buildings of the Universiti Tenaga Nasional. X. Yu et al. built up a mathematic model to estimate hydraulic transients in long diversion type hydropower station. Z. Chen et al. proposed an optimal control method for maximizing the efficiency of direct drive ocean wave energy extraction system. Y. Xu et al. investigated the iron losses in deep-sea motors when taking into account the seawater compressive stress. N. Dai et al. proposed a multifunctional voltage source inverter for renewable energy integration and power quality conditioning. A. Tomczewski presented the issues of a wind turbine flywheel energy storage system operation under real conditions. H. Yuan et al. investigated biochars derived from banana at different thermotreatment temperatures and with or without chemical activation. A. Hubackova et al. reported a solar drying technique for fish processing in Cambodia. Q. Wang et al. proposed an optimal coefficients selection method for improving power quality of photovoltaic generation. M. A. Islam et al. comprehensively reviewed various sources of renewable energy and their efficient utilization all over the world. C. Peng and K. Qian developed a ZigBee-based building energy monitoring and control system. S. Apelfröjd and S. Eriksson presented a novel electrical system configuration for variable speed wind turbines. H. Geng and G. Yang discussed the structure, performance, implementation cost, advantages, and disadvantages of different linear and nonlinear schemes applied to the pitch control of wind power generation systems.

In summary, how to tap renewable energy sources efficiently and economically remains a huge challenge for all of us. With no doubt, development of technologies in electrical and electronic engineering, control engineering, and material sciences will bring our human being to a clean and sustainable future. We look forward to further new progress on the basis of and beyond the work reported in this issue.

*Linni Jian* *Linni Jian* *Hua Bai* *Hua Bai* *Wenxiang Zhao* *Wenxiang Zhao* *Jianing Liang* *Jianing Liang*

